# Supplementary material for: High Temperature Alters Secondary Metabolites and Photosynthetic Efficiency in Heracleum sosnowskyi
Source: Int J Mol Sci. 2021 Apr 30;22(9):4756. doi: 10.3390/ijms22094756 (PMC8124411; doi:10.3390/ijms22094756)
Supplement: Supplementary file 1 [file ijms-22-04756-s001.zip › ijms-1182431-supplementary.pdf]

SUPPLEMENTARY MATERIALS

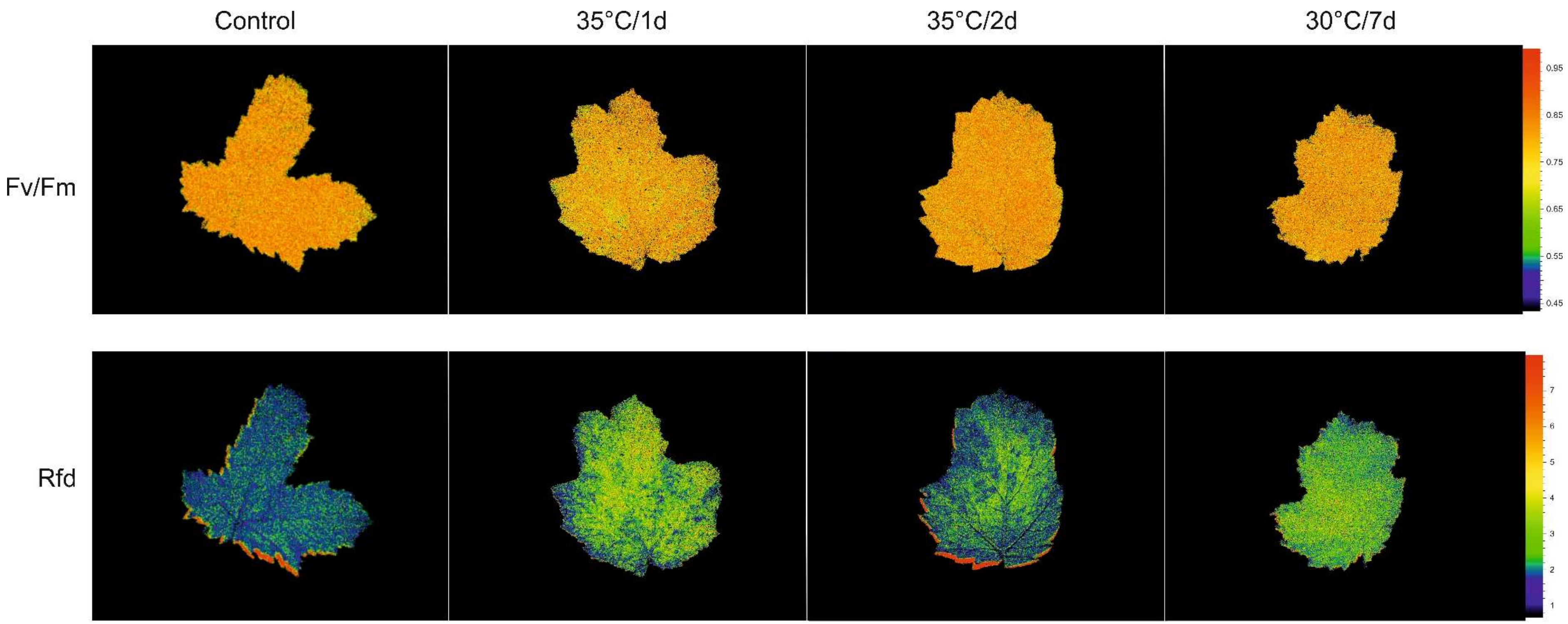

Suppl. Fig. 1. Chlorophyll *a* fluorescence parameters (Fv/Fm; Rfd) determined with an imaging PAM equipment

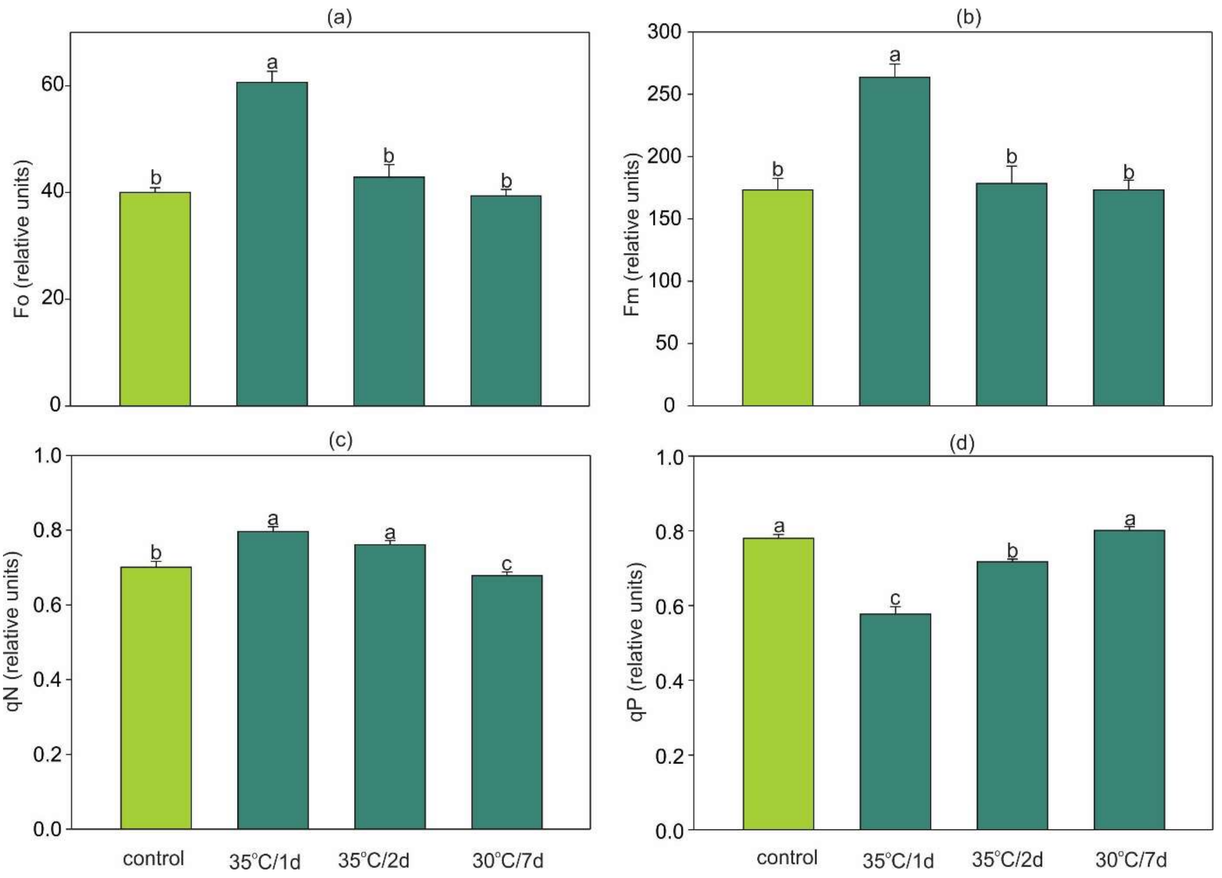

Suppl. Fig. 2. Minimal (**a**) and maximal (**b**) fluorescence in dark-adapted leaves, and the non-photochemical (**c**) and photochemical quenching (**d**) in the leaves of *H. sosnovskyi* in response to stress temperature. Data are means ± SE (n=4). Values followed by the same letter(s) are not significantly different according to Tukey's test ($p<0.05$).

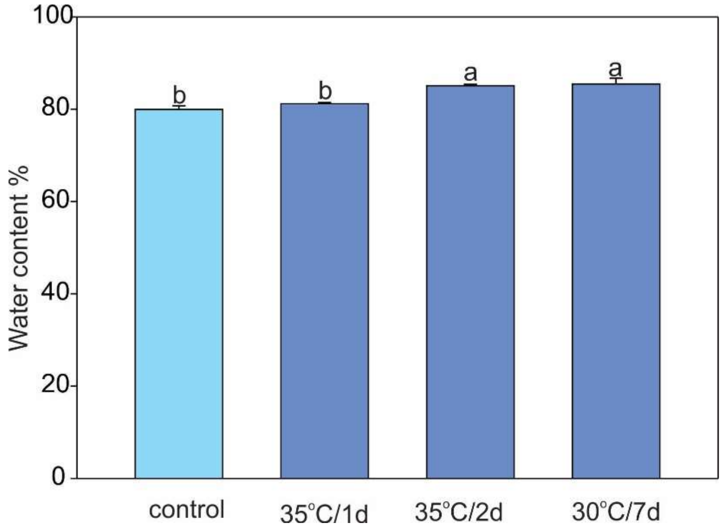

Suppl. Fig. 3. Water content in *H. sosnovskyi* leaves in response to stress temperature. Data are means ± SE (n=4). Values followed by the same letter(s) are not significantly different according to Tukey's test ($p<0.05$).

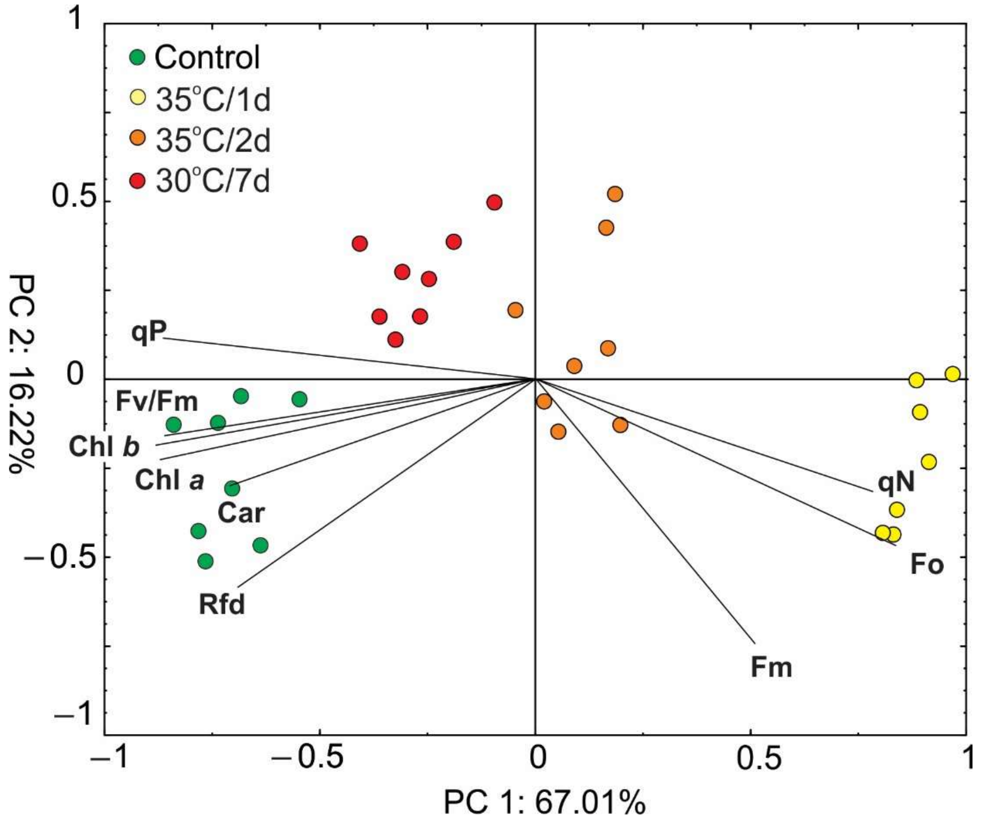

Suppl. Fig. 4. Scaled scatter plot of the principal component analysis of photosynthetic pigments (Chl *a*, Chl *b*, Car) and parameters of chlorophyll *a* fluorescence (Fv/Fm, Rfd, F_0, Fm, qN and qP). The length of lines shows a correlation between original data and the factor axes.
